# Potential Use of BEST^®^ Sediment Trap in Splash - Saltation Transport Process by Simultaneous Wind and Rain Tests

**DOI:** 10.1371/journal.pone.0166924

**Published:** 2016-11-29

**Authors:** Mustafa Basaran, Oguzhan Uzun, Wim Cornelis, Donald Gabriels, Gunay Erpul

**Affiliations:** 1 Erciyes University, Faculty of Agriculture Department of Soil Science and Plant Nutrition, Kayseri, Turkey; 2 Ankara University, Faculty of Agriculture Department of Soil Science and Plant Nutrition, Ankara, Turkey; 3 Ghent University, Department of Soil Management, International Centre of Eremology (ICE), Ghent, Belgium; Centro de Investigacion Cientifica y de Educacion Superior de Ensenada Division de Fisica Aplicada, MEXICO

## Abstract

The research on wind-driven rain (WDR) transport process of the splash-saltation has increased over the last twenty years as wind tunnel experimental studies provide new insights into the mechanisms of simultaneous wind and rain (WDR) transport. The present study was conducted to investigate the efficiency of the BEST^®^ sediment traps in catching the sand particles transported through the splash-saltation process under WDR conditions. Experiments were conducted in a wind tunnel rainfall simulator facility with water sprayed through sprinkler nozzles and free-flowing wind at different velocities to simulate the WDR conditions. Not only for vertical sediment distribution, but a series of experimental tests for horizontal distribution of sediments was also performed using BEST^®^ collectors to obtain the actual total sediment mass flow by the splash-saltation in the center of the wind tunnel test section. Total mass transport (kg m^-2^) were estimated by analytically integrating the exponential functional relationship using the measured sediment amounts at the set trap heights for every run. Results revealed the integrated efficiency of the BEST^®^ traps at 6, 9, 12 and 15 m s^-1^ wind velocities under 55.8, 50.5, 55.0 and 50.5 mm h^-1^ rain intensities were, respectively, 83, 106, 105, and 102%. Results as well showed that the efficiencies of BEST^®^ did not change much as compared with those under rainless wind condition.

## Introduction

The fundamentals of WDR erosion processes have been developed by the studies performed in the wind tunnel rainfall simulator facility at the International Centre for Eremology (ICE), Ghent University, Belgium [1–3]. Especially, some of these have well documented that splash-saltation process could cause net transportation in the prevailing wind direction [4]. This also brought new insight for the process mechanics in which particle detachment or dislodgement (splash) and particle saltation, respectively, by raindrop impact and wind, constitute major components of the transport system. This cooperative work of rain and wind for eroding soil is significantly different from those under rain-free wind and wind-free rain [5,6].

Although there are accumulated works performed under controlled laboratory conditions for essentials of WDR erosion processes, field studies and observations are still very rare [7–9] particularly for the splash-saltation transport process. The reason is that, despite the fact that a certain number of active and passive aeolian sediment traps for wind-driven sediment transport exist, there has been no trap specifically designed to catch and quantify splash-saltating particles during rainfall events accompanied by strong winds previously.

As mentioned earlier, there are several studies where passive and active traps were developed and used for the measurement of sediments transported by different wind-driven erosion processes [10–19]. Of these traps, the most common ones are BSNE (Big Spring Number Eight) and WAC (Wilson and Cooke) passive traps [20,21]. Several researchers worked with various traps by different aerodynamic designs and dimensions for actual wind erosion measurements and successfully modeled sediment flux and tried to explain both vertical and horizontal sediment transport characteristics [12,20–32]. Among those, to our knowledge, though, only a modified version of the WAC catcher was tested for the splash-saltation transport under WDR conditions in the ICE wind tunnel by [22]. However, in most of the wind tunnel and field studies on splash—saltation processes used sediment collection containers without consideration of their efficiency under WDR events [1,4,33–39].

The BEST^®^ (Basaran and Erpul Sediment Trap) [40] with cyclone-type aerodynamic counters and modular plastic bodies is newly designed trap to catch sediments transported through both saltation and suspension. The trap has efficiencies of 80–100% at different particle sizes and wind velocities. The cyclone system of the BEST provides a great advantage for trapping dust particles with a consistent efficiency. The present study was conducted to determine an integrated efficiency of BEST^®^ over a vertical height of 0.23 m in measuring splash-saltation sediment transport observed under WDR conditions.

## Materials and Method

### Wind tunnel

Efficiency tests of BEST^®^ were performed in the wind tunnel at the International Centre for Eremology (ICE) of Ghent University, Belgium. The wind tunnel has a length of 12 m and is 1.2 m wide and 3.2 m high. The wind profile within the tunnel is expressed by the Prandtl–von Kármán equation and the boundary layer thickness of the ICE wind tunnel was set at 0.61 m by a combination of spires and roughness elements [41].

For each wind velocity of 6, 9,12 and 15 m s^-1^, test WDR intensities (*I*_*wdr*_) were 55.8, 50.5, 55.0 and 55.5 mm h^-1^, respectively. This was because of different rain displacements in the limited test area of the wind tunnel; and depending on the rain inclinations from the vertical driven by different wind velocities. Because of this, it was not possible to work with a wide range of intensities. By an independent run without a sand tray before the relevant splash-saltation measurement, *I*_*wdr*_ was directly measured with small collectors on the horizontal plane for a nozzle operating pressure of 100 kPa and the horizontal wind velocities of 6, 9, 12, and 15 m s^−1^ [42–44]. That is, the collectors were placed exactly at the same location in the tunnel where the sand tray was set up. In this way, the *I*_*wdr*_ measurements were truly representative of each run without any need for correction due to the rain inclination [45,46].

### Description of BEST^®^

**Fig 1:**
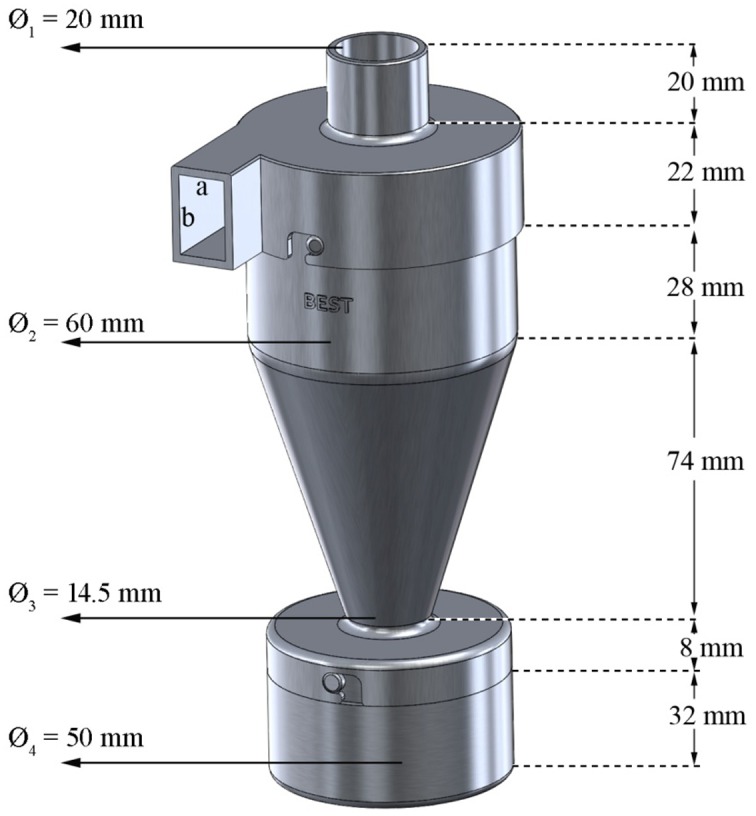
Technical drawing of the BEST^®^ with dimensions.

The BEST^®^ sediment catcher has a plastic body manufactured by a plastic injection system. It is comprised of three modular units, which are a lid including an inlet and outlet, a cylindrical cyclone body and a collector, which were designed so that they could easily be assembled and disassembled (Fig 1). Air flow characteristics and efficiencies of the trap under different rain-free wind velocities were, described in detail by [40].

### Efficiency tests

The tests of the BEST^®^ under WDR were conducted in a wind tunnel rainfall simulator with water sprayed at 100 kPa through sprinkler nozzles (Teejet TG SS 14 W nozzle from Spraying Systems Co.1, Weeton IL, USA) under 55.8, 50.5, 55.0 and 50.5 mm h^-1^ rain intensities driven by 6, 9, 12, and 15 m s^-1^ wind velocities. Wind speeds were measured by a valve-type probe located at *x* = 1.2 m, *y* = 0.6 m and *z* = 0.75 m, where *x* is the distance from the tunnel entrance, *y* is the distance from the tunnel wall, and *z* is the height above the tunnel floor. The wind velocity profiles above the sand tray were characterized by the following logarithmic equation:

$$u(z)=\left(\frac{u_{*}}{\kappa }\right)\text{ln}\left(\frac{z}{z_{0}}\right)\text{ for }z|>z_{0}$$ (1)

where *u(z)* is the wind velocity at height *z*, *z*_*o*_ is the aerodynamic roughness height, u_*_ is the wind shear velocity, and *K* is von Karman’s constant. The boundary layer was set at 0.61m above the sand tray. Subsequently, the reference shear velocities were derived from the logarithmic wind profiles, assuming a fixed roughness height of 0.0001 m for a bare and smoothed sand surface. Calculated reference shear velocities are 0.34, 0.50, 0.66 and 0.81 m s^-1^ for the reference wind velocities of 6, 9, 12 and 15 m s^-1^, respectively.

**Fig 2:**
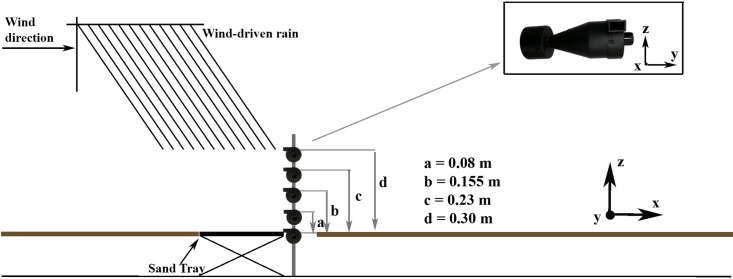
Experimental setup for the efficiency test of the BEST^®^ under the process of the splash-saltation of WDR.

**Fig 3:**
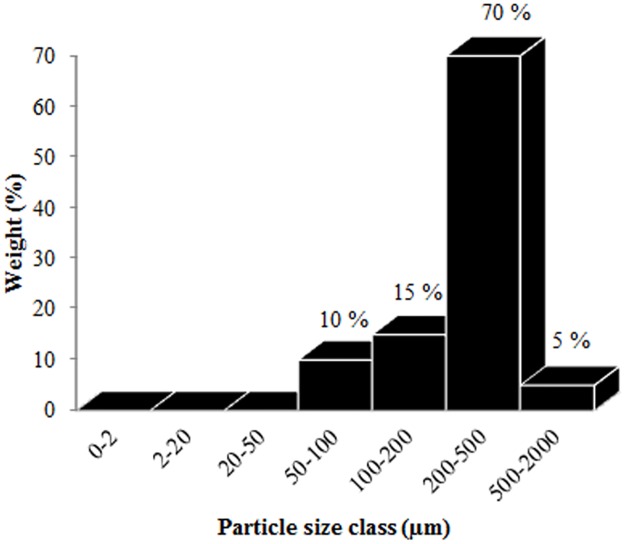
Histogram of particle-size distribution for the sand used in the WDR.

A sand tray (0.34×0.24x0.01 m) was placed at *x* = 6.5 m, *y* = 0.43 m and *z* = 0.30 m (Fig 2). The sand used in this study was collected from the Belgian coast and its particle-size distribution histogram is given in Fig 3. Geometric mean particle diameter was 250 μm. Calcium carbonate and organic matter contents were 3.3% and 0%, respectively [36]. The traps were placed horizontally on the x-axis and fixed to a mast at the heights of 0.006, 0.08, 0.155, 0.23 and 0.30 m above the tunnel floor measured from the center of the trap's inlet and at *x* = 6.74 m, i.e., immediately windward from the sand tray as shown in Fig 2. Each experiment was replicated three times and almost 1250 g sediment was placed in the sand tray per run. After each run, a total of splash-saltated sediment loss (*Q*_*l*_,*kg*) from the sand tray was determined by weighting the remaining sediment in the tray on an electronic balance with a 0.01 g precision. The collected sediments in the traps were washed into aluminum boxes for drying in an oven at 105°C for 24 h to determine the trapped amount at every height. Twelve runs were thus performed with four different wind velocities and three replications.

**Fig 4:**
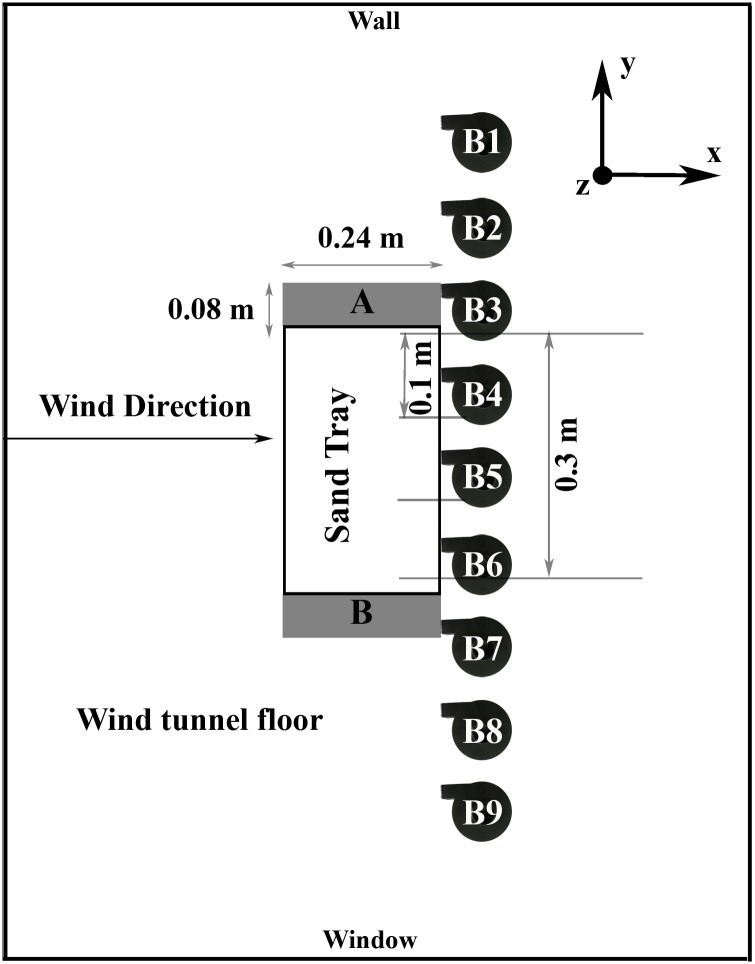
Experimental setup for determining actual amount of sediment flow passing through the center of wind tunnel (0.3 m).

Since horizontal sediment flow pattern in the wind tunnel was not perfectly homogeneous, two different corrections were performed to provide a control volume approach [47]. Firstly, the amount splashed to both sides (right and left) of the sand tray and not entered into sediment flow was deducted from the total loss to determine the actual amount of sand passing through the tunnel test section. For that purpose, splash cups were placed on the right and left sides of the sand tray (Fig 4). After each run, losses from the sand tray to the splash cups were weighted and the first correction factor (*C*_*f1*_) was determined (Eq 2).

Secondly, the amount of sediment passing through the center of the tunnel was measured and the second correction factor (*C*_*f2*_) was introduced for each wind velocity by using nine BEST^®^ traps which were horizontally placed along the width of tunnel floor from its window to its side wall with 0.10 m intervals (Fig 4). The *C*_*f2*_ enabled us to obtain actual total sediment distribution in the center of the wind tunnel test section (0.3 m), where the trap inlets were positioned. A total of twelve runs were thus additionally carried out with four different wind velocities (similar as those for the efficiency tests as described above) and with three replications to determine *C*_*f2*_ values. After each test, the trapped sediment was weighed to determine sand transport (kg m^-2^) in the center of the wind tunnel. The *C*_*f2*_ values were then calculated by Eq (3) as an average of three replicates at each wind velocity level. The *C*_*f1*_ and *C*_*f2*_ values together with their descriptive statistics such as mean, standard deviation and coefficient of variation for corresponding wind velocities are given in Table 1.

$$Cf_{1}=\frac{Q_{t}-(A+B)}{Q_{t}}$$ (2)

$$Cf_{2}=\frac{\sum_{i=4}^{6}{\left(Q_{trap}\right)}_{i}}{\sum_{i=1}^{9}{\left(Q_{trap}\right)}_{i}}$$ (3)

where *Q*_*t*_ (g) is the total loss from sand tray, A and B (g) are the amount of the collected sand by splash cups, *Q*_*trap*_ (g) is the weight of sediment trapped by the BEST^®^ traps placed from the tunnel window to its side wall (1.2 m).

**Table 1:** *C*_*f1*_ and *C*_*f2*_ values for different wind velocities.

		6 (m s^-1^)	9 (m s^-1^)	12 (m s^-1^)	15 (m s^-1^)
**C**_***f1***_	**Mean**	0.93	0.93	0.94	0.94
	**SD**	0.09	0.12	0.08	0.09
	**CV**	9.87	13.16	8.71	9.45
**C*f***_***2***_	**Mean**	0.88	0.88	0.92	0.86
	**SD**	0.12	0.13	0.6	0.5
	**CV**	13.19	15.02	6.11	5.72

CV; Coefficient of variation, SD; Standard deviation

**Fig 5:**
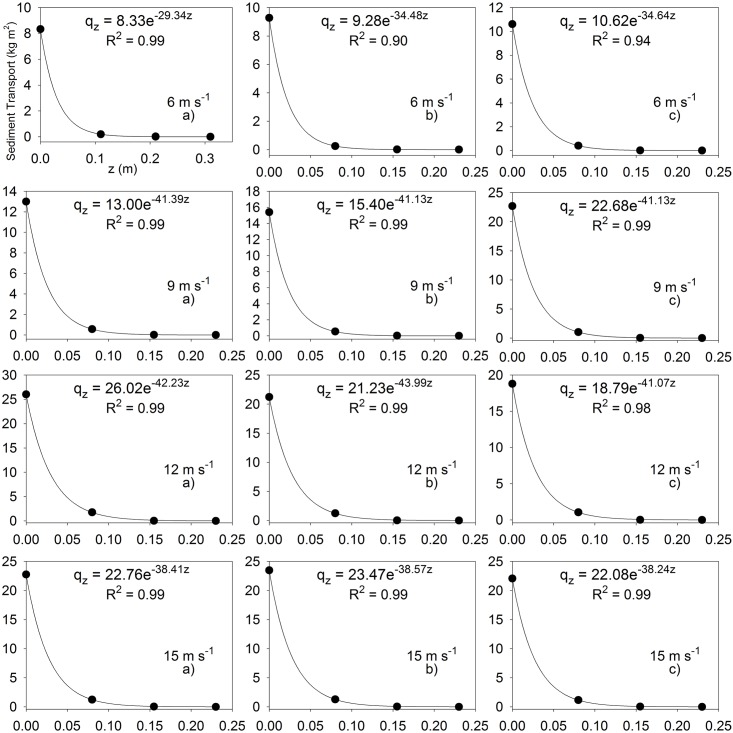
Relationships between sediment transport (kg m^-2^) and height (m).

Sediment transport (*Q*_*r*_, kg m^−2^) (Eq 4) was estimated by analytically integrating the exponential functional relationship (Eq 5) between the measured sediment weight and trap heights of 0.006, 0.08, 0.155 and 0.23 m for each run:

$$Q_{r}=\int_{0}^{h}q_{z.\text{exp}}dz$$ (4)

where, *h (m)* is the maximum particle transportation height. Although the traps were positioned horizontally at the heights of 0.006, 0.08, 0.155, 0.23 and 0.30 m above the tunnel floor, the transport did not occur at the height of 0.30 m for all tests, leading to *h* = 0.23 m. *q*_*z*. *exp*_ (kg m^−2^) is the amount of sediment per trap inlet area at the set height of the *z* (m):

$$q_{z.\text{exp}}=q_{o}e^{-\alpha z}$$ (5)

where, *q*_*0*_ (kg m^-2^) is the amount of the sediment modeled at *z* = 0.006 m and α is the slope factor of the exponential regression equation (m^-1^). For using Eq (5) in the analytical integration, the amount measured at the lowermost trap was assumed to be equal to *q*_*0*_. This amount of theoretical zero height at the surface of sand tray (*q*_*0*_) determined the intercept of q-axis, i.e. the uppermost boundary of the vertical mass distribution curves (Fig 5).

With a *L* = 0.3 m wide section of the wind tunnel center (Fig 4), the total amount of the trapped sediment (*Q*, kg) was computed by:

$$Q=Q_{r}\times L$$ (6)

Trap efficiency computations were performed over the total sediment weight (*Q*_*l*_, kg) lost from the sediment tray after they were corrected by the related *C*_*f*_ values from Table 1 for each run:

$$Q_{c}=Q_{l}\times Cf_{1}\times Cf_{2}$$ (7)

where, *Q*_*c*_ (kg) is the corrected sediment weight transported from the sediment tray by the splash—saltation process. Dimensionless trap efficiencies of the BEST^®^ traps were calculated by a ratio of *Q* over *Q*_*c*_:

$$\eta =\frac{Q}{Q_{c}}$$ (8)

Along with the trapezoidal model, the Riemann Sum Middle Point calculation method was used for integrating the exponential functional relationship [48].

Clearly, this integration involved in data collection from the traps at different heights above the surface, with each experienced different splash-saltation transport depending upon the mean wind velocity profile within the tunnel. With this research set-up, it is significant to note that an integrated efficiency calculated from vertically integrated splash-saltation sediment transport across a vertical plane perpendicular to the flow up to the height of 0.23 m was obtained experimentally.

## Results and Discussions

The relationship between sediment transport and height (vertical mass distribution curves *q*_*0*_ = *qz*_*1*_ (kg m^-2^), where *z*_*1*_ = 0.006 m) under WDR conditions is graphed in Fig 5. Sediment transport exhibited an exponential decrease with height at each wind velocity, with the majority of the sediment moving within the first centimeters from the sand surface with these velocities. Similarly, in wind erosion studies, researchers reported that under rainless conditions almost 50% of the mass flow was observed near the soil surface [49–53]. Basaran et al. [40] investigated the efficiency of the BEST sediment trap under rainless condition, working with the same sand and similar wind velocities as those of this research. When the findings of both studies were compared, to some extent, different vertical trajectories of sand particles were found for the wind velocities of 12 and 15 m s^-1^. For example, the maximum vertical trajectory of sand particles was 0.2–0.25 m and sediment transport mostly occurred in the first 0.05 m in this study of WDR while they were 0.4–0.5 m and 0.3 m, respectively, under rainless condition [40]. Erpul et al. and Cornelis et al. [4,23] indicated that sand particles were lifted off by the wind-driven raindrop impact, transported some distance within droplets by wind streams during the splash-saltation transport process described as a combined operation of raindrop and wind. The researchers stated that the maximum vertical trajectory and the sediment transport height in the process could be lower than those of only wind-driven process of saltation because of increased gravity forces of sand particles encapsulated in the droplets compared to individual dry sand particles. Also re-distribution of droplets driven by wind could decrease the maximum vertical trajectory and the sediment transport height.

Efficiencies of the BEST^®^ sediment traps in catching sands transported through splash-saltation at the wind velocities of 6, 9, 12, 15 m s^-1^ are provided in Table 2.

**Table 2:** Efficiencies of the BEST^®^ traps (%) for the splash—saltation process of WDR.

	Wind velocity (m s^-1^)			
	6	9	12	15
**Mean**	83	106	105	102
**SD**	5.93	0.47	11.00	0.24
**CV**	7.14	0.44	10.51	0.23

SD; Standard deviation, CV; Coefficient of variation

The efficiency values were 83, 106, 105, and 102%, respectively, for the wind velocities of 6, 9, 12, and 15 m s^-1^. Small variations in the efficiencies with wind velocities might be due to uncontrolled random variations. For instance, different free stream wind flows were formed during the four wind velocity measurements in the tunnel. The differences in free wind flow streams in each replications might have resulted in a small increase or decrease in efficiency values. Greater standard deviation and variation coefficients were found at 6 and 12 m s^-1^ wind velocities (5.93–11.00 and 7.14–10.51 m s^-1^, respectively) indicated that the measurements made at these wind velocities were affected, in some degree, by uncontrolled measurement conditions within the wind tunnel. Variation of wind turbulence, little changed by the intensity of simulated rainfall and measurement errors could lead to a greater standard deviation and a variation coefficient.

The experimental set-up could also affect the trap efficiencies. Especially, a closer spacing of the traps to each other on the mast, could lead to some degree of stagnation pressure and a decreased trapping efficiency. The static pressure problem at trap inlet also was dealt with in the trap used by [54].

As stated previously, there is no particular efficiency study designed to capture particles of the splash-saltation process in WDR with passive traps although many wind tunnel studies have been done to measure wind-driven saltation without rain. However, in those tests, specifically in terms of trap number used to catch the particles either at a certain height (one-height trap measurements) or across a vertical plane (multi-height trap measurements) perpendicular to the flow, there is no a single standard procedure for determining efficiency. Assuming highly controlled conditions and homogeneous sediment flow at different heights in a test tunnel, some researchers carried out one-trap experiments for efficiency. The efficiency tests for saltation with the commonly used BSNE and WAC traps were used under completely different physical conditions of both air flow and particle characteristics. For example, Fryrear [20] conducted a one-trap experiment with BSNE using three different grains (sand, sieved soil and washed sand) and three different wind velocities of 10.4, 13.0 and 15.7 m s^-1^, and reported that the efficiency varied between 88 and 94% showing a tendency to decrease as wind velocity increased. Sterk [55] tested the efficiency of the WAC traps with a range of wind velocities from 9.9 to 11.5 m s^-1^ in a wind tunnel and found an efficiency of 49%, without changing with wind velocity increase, which could be, to a large extent, attributed to the narrow velocity range used in the experiment. The efficiencies of the BSNE and MWAC (with dimensions of the bottle, inlet and outlet tubes modified) traps were studied and compared at low wind velocities (1, 2, 3, 4 and 5 m s^-1^) using silt sized loess [56]. For comparative testing, regardless of the traps’ inlet dimensions, traps were positioned at the height of 23.5 cm from tunnel floor. The efficiencies of BSNE and WAC traps ranged between 35 and 45 and 75 and 90%, respectively. These results obtained were under relatively lower wind velocities with the BSNE and WAC traps and were significantly different from those under higher wind velocities [30,54]. Once again, an another study [57] compared the efficiencies of BSNE and WAC traps, under the wider ranges of wind velocity and grain size (sand), (6.6, 8.4, 10.5, 12.5 and 14.4 m s^-1^ and 132, 194 and 287 μm, respectively) and found that the efficiencies of BSNE and WAC traps ranged between 80 and 100%, concluding that both were the most efficient traps for capturing sand sized particles. This literature review shows that in wind tunnel experiments a set-up with one-height trap measurements, presumably with a trap positioned under the boundary layer thickness and within the free stream using BSNE and WAC sediment traps that both traps had changing efficiencies depending on the particle size and wind velocity. However, for their efficiency experiments, [40,58] used a set up with multi-height trap measurements with traps placed along the boundary layer thickness below the free-flowing stream using both BEST and WAC traps, respectively. In these tests, the researchers additionally did a vertical calibration to measure horizontal sediment fluxes at different set heights. Youssef et al. [58], keeping wind velocity constant at 13.4 m s^-1^, performed an efficiency study with WAC traps using five different grain sizes (<50, <75, 50–75, 200–400 and 400–500 μm) in the ICE wind tunnel. For the range of relatively much smaller grains, the efficiencies were considerably as low as 0, 0 and 14.5% for <50, <75 and 50–75 μm, respectively, and varied between 24.8 and 37.8% for the grain sizes >200 μm. These results were quite comparable with those of [55] for larger grains. Eventually, the researchers stated that the WAC traps might be successfully used for saltation grains greater than 200 μm but not efficiently usable for suspended grains lesser than 75 μm. In the cases of multi-height trap measurements, the efficiency was calculated as an average value of function over interval, it was closely linked to the boundary layer thickness or the wind velocity profile in the wind tunnel.

In this study, the BEST^®^ traps overall had a very high mean efficiency (99%) calculated by the exponential middle point. The centrifuge impact created within the BEST^®^ traps by the cyclone system possibly decreases the static pressures at trap inlets. Effects of the aerodynamic shape of the BEST^®^ traps reduces static pressures, variation with wind velocities at both trap inlets and outlets. Wind velocity acceleration within the trap and operation of cyclone system were all explained in detail by [44]. Cortés and Gil [59] stated that the aerodynamic design of the cyclone system created a centrifugal effect within the trap and decreased static pressure at the entrance. It was explained by previous researchers that static pressures at trap inlets had the greatest impacts on efficiencies of the WAC and BSNE traps commonly used in wind erosion measurements [30,57]. In BEST^®^ traps, a cyclone design allows a pressure difference between the inlet and outlet, which sufficiently draws the flow into trap and prevents static pressures and ultimately allows more sediment that gets into it to be caught by the trap. Another advantage of the BEST trap was the larger inlet diameter which was 240 mm^2^. A larger trap inlet could also facilitate readily trapping of big particles and droplets. Cornelis et al. [23,60] increased the inlet diameter of the WAC catcher from 0.8 to 2 cm to prevent plugging of the trap inlet and consequently, the efficiency of MWAC was calculated as 40%.

For the first time, different from the previous wind erosion studies, the BEST^®^ traps were placed horizontally on a mast in this study. High efficiency values indicated that the BEST^®^ sediment traps could successfully be used horizontally in wind erosion measurements, as well. Horizontal installations would then allow the researchers to take measurements at relatively small intervals from the soil surface. This could improve the collection of data for the modeling efforts and reduce estimation errors.

## Conclusions

Comprehension of splash-saltation mechanics under WDR conditions could only be possible through proper determination of the maximum horizontal and vertical transportation distances, average horizontal and vertical transportation distances and the forces affecting these parameters. The results revealed that the BEST traps could reliably be used as an alternative to classical sediment collection devices and could conveniently be used as a new tool in detailed measurements of the splash-saltation process.

Relatively high efficiency values of the BEST traps at horizontal position in addition to those at the vertical position potentially increased their usability to trap sediments transported through processes of both rain-free wind erosion and WDR splash-saltation.

## Supporting Information

S1 File Raw Data. (XLSX)
